# Evaluation of Inhibitory Antibodies against the Muscarinic Acetylcholine Receptor Type 3 in Patients with Primary Biliary Cholangitis and Primary Sclerosing Cholangitis

**DOI:** 10.3390/jcm11030681

**Published:** 2022-01-28

**Authors:** Anne-Christin Beatrice Wilde, Lena Maria Greverath, Lara Marleen Steinhagen, Nina Wald de Chamorro, Elise Leicht, Janett Fischer, Toni Herta, Thomas Berg, Beate Preuss, Reinhild Klein, Frank Tacke, Tobias Müller

**Affiliations:** 1 Department of Hepatology and Gastroenterology, Campus Virchow Klinikum, Charité—Universitätsmedizin Berlin, 13353 Berlin, Germany; lena-maria.greverath@charite.de (L.M.G.); lara.steinhagen@outlook.de (L.M.S.); nina.wald-de-chamorro@charite.de (N.W.d.C.); elise.leicht@gmail.com (E.L.); frank.tacke@charite.de (F.T.); tobias.mueller@charite.de (T.M.); 2 Division of Hepatology, Department of Medicine II, Leipzig University Medical Center, 04103 Leipzig, Germany; janett.fischer@medizin.uni-leipzig.de (J.F.); toni.herta@medizin.uni-leipzig.de (T.H.); thomas.berg@medizin.uni-leipzig.de (T.B.); 3 Department of Internal Medicine II, University of Tübingen, 72076 Tübingen, Germany; beate.preuss@med.uni-tuebingen.de (B.P.); reinhild.klein@med.uni-tuebingen.de (R.K.)

**Keywords:** biliary bicarbonate umbrella, chronic biliary inflammation, muscarinic acetylcholine receptor type 3, primary biliary cholangitis, primary sclerosing cholangitis, ursodeoxycholic acid

## Abstract

Background: Primary biliary cholangitis (PBC) and primary sclerosing cholangitis (PSC) constitute rare chronic inflammatory biliary diseases which likely comprise genetic, environmental and autoimmune factors. Specific inhibitory (auto-) antibodies against the muscarinic acetylcholine receptor type 3 (mAChR3 auto-ab) may contribute to the pathogenesis of chronic biliary inflammation by modulating mAChR3− mediated signaling. Aims: The aim of this study was to analyze the prevalence and relevance of inhibitory mAChR3 auto-ab (mAChR3inh+ auto-ab) in a large cohort of PBC patients from two independent tertiary centers in Berlin and Leipzig in comparison to a large PSC cohort. Baseline parameters and response rates to standard treatment with ursodeoxycholic acid (UDCA) were characterized with respect to the individual mAChR3 auto-ab status. Methods: In total, the study population comprised 437 PBC patients, 187 PSC patients and 80 healthy controls. Clinical and laboratory baseline characteristics were retrieved from medical records. The response to ursodeoxycholic acid (UDCA) therapy after 12 months of treatment was available in 176 PBC and 45 PSC patients. Results: The prevalence of mAChR3inh+ auto-ab was significantly higher among PBC patients (11.2%, 49/437; *p* = 0.008 vs. healthy controls) and PSC patients (33.6%, 63/187; *p* < 0.0001 vs. healthy controls) compared to healthy controls (2.5%, 2/80), respectively. PBC patients with mAChR3inh+ auto-ab exhibited significantly higher levels of alkaline phosphatase (ALP) and bilirubin, which constitute established parameters for PBC risk stratification. Moreover, mAChR3inh+ PBC patients tended to show decreased response rates to UDCA therapy compared to PBC patients without mAChR3inh+ auto-ab (mAChR3− PBC). In contrast, PSC patients with mAChR3inh+ auto-ab showed no significant differences in laboratory findings compared to mAChR3 auto-ab negative (mAChR3−) PSC patients. Conclusion: MAChR3inh+ auto-ab might be involved in the pathogenesis and treatment response of chronic biliary inflammation in patients with PBC but not in patients with PSC.

## 1. Introduction

Primary biliary cholangitis (PBC) and primary sclerosing cholangitis (PSC) are rare chronic inflammatory biliary diseases with increased morbidity and mortality [1,2]. There is growing evidence suggesting the involvement of multiple genetic, environmental, microbiological and especially autoimmune factors contributing to disease development [1,2]. Moreover, affected biliary epithelial cells (BECs) are considered to be actively involved in the progression of chronic biliary inflammation [3].

In all cholangiopathies, BECs represent the first line of immune defense, e.g., by expressing a number of receptors capable to recognize pathogen- or damage-associated molecular patterns. Accordingly, these cells can be the target and initiator of inflammation [4,5]. Furthermore, the so-called protective biliary bicarbonate umbrella [6,7,8] has been proposed to play a crucial role in the hepatobiliary acid-base homeostasis [9].

In the concept of the biliary bicarbonate umbrella, Beuers et al. and Hohenester et al. proposed that glycin conjugated bile acids, which comprise the majority of bile acids, diffuse passively across the luminal cell membrane of the BECs and have potential cholangiotoxic effects by inducing apoptotic signaling cascades. The secretion of high concentrations of bicarbonate by the BECs induces an alkaline environment near the apical cell membrane in which the bile acids are deprotonated and lose their membrane permeability in this hydrophilic charged form. Consequently, this protects the BECs from bile acid toxicity [6,7].

However, continuous exposure to exogenous triggers may lead to functional anti-receptor auto-ab that are not protective but rather deleterious [10].

In other autoimmune diseases, a functional relevance of detectable auto-ab has already been established. For example, in Sjögren’s syndrome (SS) [11] auto-ab against the muscarinic acetylcholine receptor type 3 (mAChR3) with inhibitory function [12,13,14] are causative of disease manifestation as the receptor plays a critical role in exocrine secretion of the salivary gland epithelia [15]. Furthermore, mAChR3 knock out mice showed significantly less tear volume, suggesting that auto-ab against the mAChR3 play a critical role in the pathogenesis of the dry eye syndrome [16]. 

This is particularly relevant as PBC is often associated with Sicca and Sjögren’s syndrome with a co-incidence of up to 40% [17,18,19]. Of note, the mAChR3 is not only expressed in salivary glands but also in cholangiocytes [15]. Importantly, the receptor is exclusively expressed on the bile duct epithelium and not in hepatocytes [20,21]. Both diseases (PBC and Sjögren’s Syndrome) are chronic organ-specific autoimmune diseases with similar underlying pathomechanisms [18]: Apoptosis as the major cellular process leads to generation of autoantigens and results in a T-cell- and later in a B-cell immune response [18]. Overall, apoptosis leads to organ-specific immune-mediated injury of epithelial cells (BECs and salivary gland epithelia) [18]. Consequently, both diseases can be considered as chronic autoimmune epithelitis [18]. In addition, there are similar epidemiological factors such as the predominance of the female sex and the onset of the disease in the fifth decade of life [18,19].

In cholangiocytes, activation of basolateral mAChR3 by acetylcholine induces an increase in intracellular calcium concentration via an increase in intracellular inosylphosphate-3 (IP3) concentration. This leads to an increase in chloride efflux via activation of cAMP and calcium chloride transport and, thus, to secretion of bicarbonate into the bile duct lumen via activation of anion exchanger 2 [22]. Subsequently, blockade of the mAChR3 due to inhibitory auto-ab might lead to reduced bicarbonate secretion and therefore to chronic inflammation due to less protection of toxic bile acids through the loss of the biliary bicarbonate umbrella [6,7,8].

Therefore, it is conceivable that auto-ab against mAChR3 may play a critical role in the pathogenesis of inflammatory bile duct diseases such as PBC or PSC. 

In line with this hypothesis, in clinical studies the presence of mAChR3 auto-ab was found in up to 90% of sera from PBC patients [23,24]. Furthermore, in an experimental mouse model genetic loss of the mAChR3 gene with altered receptor function led to reduced biliary bicarbonate secretion and consecutive cholangiopathy [25]. Moreover, muscarinic acetylcholine agonists such as bethanechol have been shown to reduce hepatic injury in this mouse model [25]. 

We were able to show a potential inhibitory function of these mAChR3 auto-ab in vitro [26] and, moreover, we have recently shown that isolated mAChR3inh+ auto-ab inhibit mAChR3 function in BECs in vitro [27]. 

Taken together, there is increasing evidence that functional mAChR3 auto-ab may contribute to the pathogenesis of chronic biliary inflammation. Therefore, the aim of this study was to analyze the prevalence of functional mAChR3 auto-ab in two large and independent cohorts of patients with PBC from two different tertiary centers and a large cohort of PSC patients. Moreover, the relation between mAChR3 auto-ab and the patients’ clinical characteristics and treatment response to standard UDCA as well as the long-term outcome of these patients was evaluated.

## 2. Materials and Methods

### 2.1. Study Population

The present study was approved by the Ethics Committees of Medical Research of the University of Berlin (reference numbers EA2/035/07; 03-2015 EA2/095/18) in accordance with the Declaration of Helsinki from 1975, and written informed consent was obtained from all patients. 

In total, 437 patients with PBC from the Department for Hepatology and Gastroenterology of Charité University Hospital Berlin and the Department for Hepatology of University Hospital Leipzig and 187 patients with PSC who were treated in Berlin were evaluated for inclusion. 

The diagnosis of PBC was accepted if patients fulfilled at least two of the following criteria: (1) chronic cholestasis for >6 months; (2) positivity for AMA, titer >1:40, or positivity for specific antinuclear ab immunofluorescence or enzyme-linked immunosorbent assay results (sp100, gp210), if AMA were negative; and (3) PBC specific liver biopsy, if PBC-specific ab were absent, as previously described in detail [21,28,29]. Concomitant features of autoimmune hepatitis were defined according to the current European Association for the Study of the Liver PBC treatment guidelines and were histologically confirmed [28]. 

The diagnosis of PSC was accepted if patients fulfilled the following criteria: (i) laboratory findings consistent with chronic cholestasis; (ii) characteristic imaging proven by magnetic resonance cholangiopancreaticography or endoscopic cholangiography; and (iii) no evidence of secondary sclerosing cholangitis, as described in detail [30]. The diagnosis of overlapping AIH was accepted if patients fulfilled the criteria according to the current clinical practice guidelines of the European Association for the Study of the Liver [31]. Patients with concurrent features of biliary obstruction, drug-induced cholestatic liver disease, nonalcoholic fatty liver disease, hemochromatosis, Wilson’s disease, alpha1-antitrypsin deficiency, alcohol abuse, and chronic hepatitis B or hepatitis C were excluded by extended laboratory testing and imaging, including abdominal ultrasound and magnetic resonance cholangiopancreatography.

### 2.2. Prevalence of Functional mAChR3 Auto-ab in PBC and PSC Patients

Sera from all 437 PBC patients as well as from all 187 PSC patients were analyzed for the presence of functionality of mAChR3 auto-ab. Serum samples were stored at −20 °C. Immunoglobulins from patient’s sera were isolated using ammonium sulphate precipitation and subsequently stored at −20 °C. Detection of functional auto-ab against mAChR3 was performed as previously described by our group [26]. In short, Chinese hamster ovary cells (CHO) stably transfected with a calcium-sensitive bioluminescent fusion protein were transiently transfected with a full-length mAChR3 plasmid DNA. After an incubation period of 24 h the cell culture was incubated with patients’ immunoglobulins for another 24 h. A titer of 1:100 was used for optimal result, which was established prior to our work (unpublished data). After adding CaCl_2_ and carbachol, changes in intracellular Ca^2+^ was measured by emitted luminescent signal. The range of normal values was analyzed by receiver operating curves (ROCs) in comparison with healthy controls. A luminescent signal with <80% relative luminescene units (RLU) in comparison with healthy individuals was defined as lower limit of normal, meaning the function of the mAChR3 was decreased after adding immunoglobulins. Those cases were determined as positive for inhibitory auto-ab against mAChR3 (mAChR3inh+). Individuals with a luminescent signal >120% were assessed as excitatory auto-ab. Patients with >80% RLU were defined as mAChR3−. 

Since only a few patients had mAChR3stim+ auto-ab, we focused our further analyzes on the clinical relevance of mAChR3inh+ auto-ab, which were found in a larger proportion of patients. All measurements were validated by positive and negative controls.

Moreover, measurements were performed in quadruplicates. Results are given in mean values. Analyses of controls showed an intra-assay variability of 20% and inter-assay variability of around 25%. Sera from all patients and healthy controls were stored at −20 °C. For purification of immunoglobulins and final measurements, samples were thawed twice on ice.

### 2.3. Baseline Characteristics

Baseline characteristics comprised sex, age at time of diagnosis, laboratory parameters (serum levels of alanine aminotransferase (ALT), aspartate aminotransferase (AST), alkaline phosphatase (ALP), γ-glutamyltransferase (γGT), bilirubin, albumin, platelet count, anti-mitochondrial ab (AMA), anti-smooth muscle ab (SMA), anti-nuclear ab (ANA), anti-sp100, anti-gp210) comorbidities and, if available, histological evaluation, ultrasound and elastography measurements at time of diagnosis of PBC of PBC. We compared histological disease stages of mAChR3− PBC and mAChR3inh+ PBC patients before initiation of therapy according to Ludwig et al. [32]. The cohort was divided into two groups (stage 3 or 4 versus stage 1 or 2). Additionally, in order to be able to evaluate the treatment response of the patients after 1 year under UDCA therapy between the mAChR3inh+ and mAChR3− patients, disease stage of the patients was characterized upon ultrasound-based detection of fibrosis with absence of fibrosis classified as early disease stage and clear signs of fibrosis classified as advanced stage.

The UK-PBC Score was determined as a prognostic parameter for PBC patients [28].

The Amsterdam–Oxford Score [33], a well-established prognosis score, was determined for PSC based on age, ERCP findings and laboratory parameters.

### 2.4. Evaluation of Treatment Response to Standard UDCA Therapy

If available, well-established laboratory values were evaluated at 12 months after treatment initiation according to Paris I, Paris II, Barcelona and Rotterdam criteria and ALP normalization [28] based on their mAChR3 auto-ab status in patients with PBC.

### 2.5. Statistical Analysis

All statistical analyses were performed with SPSS software (Version 25.0 SPSS for Windows; IBM Corp., Armonk, NY, USA). Data are presented by median and interquartile range (IQR) unless stated otherwise. Mann–Whitney test, Fischer’s exact test and Kruskal–Wallis test were performed for comparison of the groups. The LogRank test was examined for statistical assessments of survival rates. A *p*-value less than 0.05 was considered as statistically significant.

## 3. Results

### 3.1. Study Population

**Figure 1:**
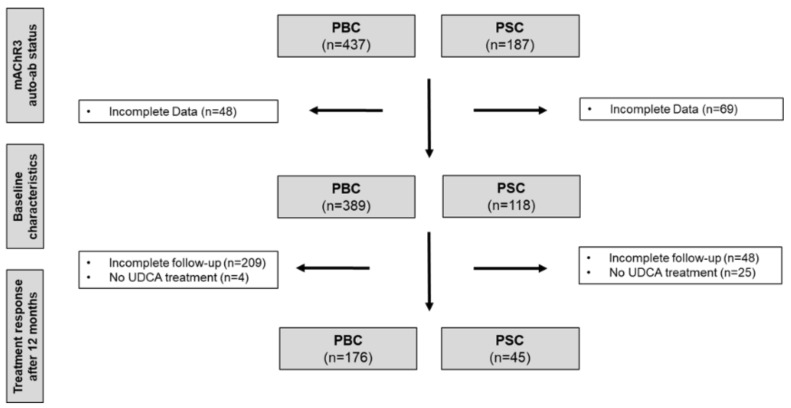
Study flow diagram. In total 437 patients with primary biliary cholangitis (PBC) and 187 patients with primary sclerosing cholangitis (PSC) from two independent fulfilled all diagnostic criteria and were included in the study.

Figure 1 shows the flow chart of the study population. In total the study population comprised 437 patients with PBC and 187 patients with PSC. Clinical and laboratory data from 389 patients with PBC and 118 patients with PSC were analyzed. A number of 48 patients with PBC and 69 patients with PSC were excluded for further analysis, mainly due to incomplete data sets. 

### 3.2. Prevalence of Functional mAChR3 Auto-ab in PBC and PSC

**Figure 2:**
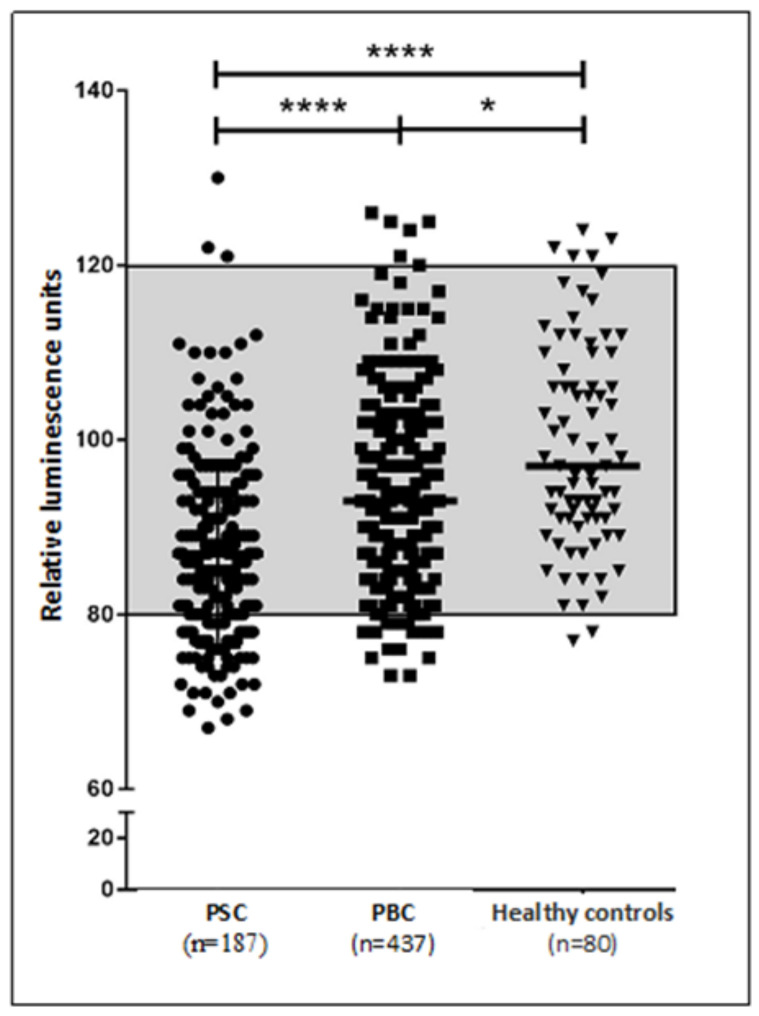
Prevalence of functional mAChR3 auto-ab in 187 PSC patients, 437 PBC patients and 80 healthy controls. A range between 80 and 120 calcium-induced relative luminescence units (RLU) was defined as normal. An RLU < 80 was defined as inhibitory effect due to mAChR3inh+ auto-ab. An RLU > 120 was defined as stimulatory effect due to mAChR3stim+ auto-ab. * *p* < 0.05, **** *p* < 0.0001; Analysis was done using Kruskal–Wallis test.

PBC: In 11.2% (49/437) of patients with PBC mAChR3inh+ auto-ab (defined as RLU < 80) could be detected which was significantly higher than in healthy controls, in whom 2.5% (2 out of 80) were positive for mAChR3inh+ auto-ab (*p* = 0.008). Stimulating auto-ab (mAChR3stim+, defined as RLU > 120) could be detected only in a few patients with 1.1% (5/437) in the PBC cohort and 6.25% (5/80) in healthy controls (Figure 2).

PSC: MAChR3inh+ auto-ab were detected in 33.7% (63/187) of patients suffering from PSC which was significantly higher as compared to patients with PBC (*p* < 0.0001) as well as to healthy controls (*p* < 0.0001). Similar to the PBC cohort, stimulating auto-ab (mAChR3stim+, defined as RLU > 120) were present only in a few patients with PSC 1.6% (3/187) (Figure 2).

### 3.3. Relation between mAChR3 Auto-ab Status and Clinical and Laboratory Baseline Characteristics and Histological Stage of Patients

PBC: The study population of 389 patients with PBC comprised 358 females (92%) of whom 11.7% (42/358) were mAChR3inh+ and 31 males (8%) of whom 12.9% (4/31) were mAChR3inh+. The median age of the population was 58 (mAChR3inh+ group) and 55 years (mAChR3− group), respectively (Table 1). AMAs could be detected in 75% (24/32) of mAChR3inh+ patients and in 75.4% (193/256) of mAChR3− patients, with no difference in the prevalence between both groups. Regarding the clinical baseline characteristics, no significant differences between mAChR3inh+ and mAChR3− patients were found in the PBC cohort (Table 1). 

**Figure 3:**
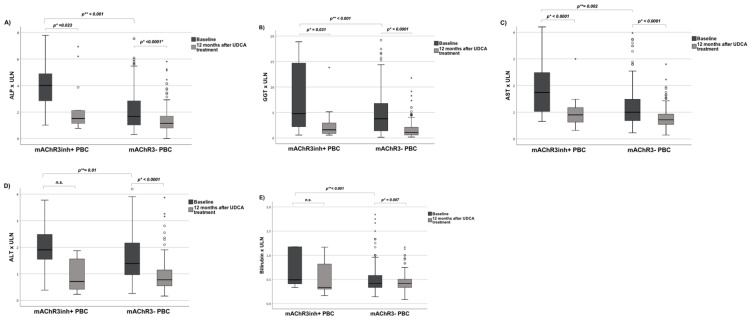
Treatment response in PBC patients 12 months after treatment initiation with UDCA according to functional mAChR3 auto-ab status. (**A**) alkaline phosphatase (ALP), (**B**) gamma glutamyltransferase (GGT), (**C**) bilirubin (**D**) Aspartate aminotransferase (AST), (**E**) Alanine aminotransferase (ALT) according to mAChR3 auto-ab status. ULN, upper limit of normal, p* = Analysis was done using Student’s *t*-Test, p** = Analysis was done using Mann–Whitney U Test.

In contrast, there were significant differences with regard to the laboratory parameters (Table 1, Figure 3). PBC patients with mAChR3inh+ auto-ab exhibited a median baseline ALP 4-times higher than the upper limit of normal (ULN) and a GGT 5.9-times higher than ULN. PBC patients without mAChR3inh+ auto-ab had an ALP 1.8-times higher than ULN (*p* < 0.0001) and a GGT only 3.4-times higher than ULN (*p* < 0.0001). MAChR3inh+ PBC patients exhibited significantly higher values of ALT, AST and total bilirubin compared to mAChR3− PBC at baseline (*p* = 0.01, *p* = 0.002 and *p* < 0.0001 respectively) (Table 1, Figure 3). 

Moreover, mAChR3inh+ PBC patients tended to be in a more advanced stage of the disease than mAChR3− PBC patients according to the histological grade classification of Ludwig et al. [32] (7/11, 63.6% vs. 41/106, 38.7%), elastography measurement (8.5 kPa vs. 8.0 kPa) and according to ultrasound analysis (13/20, 65% vs. 120/242, 49.6%) (Table 1). Furthermore, there were no differences between patients with mAChR3inh+ PBC and mAChR3− PBC regarding comorbidities (Table 1).

PSC: A total of 118 patients with PSC were included for further analysis in the study comprising 43 females (36%) of whom 27.9% (12/43) were mAChR3inh+, and 75 males (64%) of whom 44% (33/75) were mAChR3inh+ (Table 2). 

There were no differences in the clinical and laboratory baseline parameters between patients with mAChR3inh+ auto-ab versus patients without these inhibitory auto-ab (Table 2). Patients within the mAChR3inh+ group showed at baseline 3-times elevated ALP and 4.9-times elevated GGT. AST and ALT were moderately elevated. With respect to intrahepatic and extrahepatic manifestation, there was no significant difference between mAChR3inh+ compared to mAChR3− PSC patients. 

The degree of liver fibrosis in patients did not differ significantly between mAChR3inh+ and mAChR3− group based on elastography measurements, ultrasound and histological analyses (Table 2).

Regarding comorbidities, overlap syndrome with AIH was more frequent in mAChR3inh+ patients as compared to mAChR3− patients (22.2% vs. 2.7%; *p* = 0.001) (Table 2).

### 3.4. One-Year Treatment Response to UDCA in Correlation with Prevalence of Auto-ab in PBC and PSC

Figure 3 (Supplementary Table S1) shows the laboratory parameters of the PBC cohort and Supplementary Table S1 depicts the parameters of the PSC cohort 12 months after treatment initiation. The median UDCA dosage was similar in mAChR3inh+ vs. mAChR3− patients.

**Figure 4:**
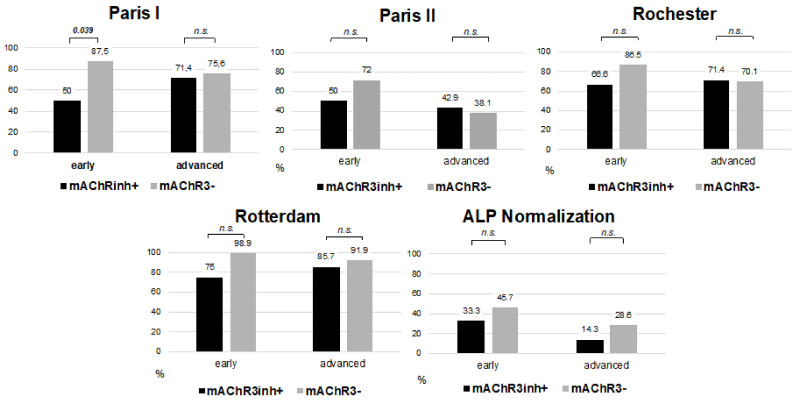
Comparison of treatment response in mAChR3inh+ and mAChR3− PBC patients 12 months after initiation of UDCA therapy according to the Rochester criterion (AP ≥ 2 × ULN or Mayo-Score ≥ 4.5), Rotterdam criterion (Bilirubin ≥ 1 × ULN and/or Albumin < 1 × ULN), Paris II criterion (ALP ≥ 1.5 × ULN or AST ≥ 1.5 × ULN or Bilirubin >1 mg/dL), Paris I criterion (ALP ≥ 3 × ULN or AST ≥ 2 × ULN or Bilirubin >1 mg/dL) and ALP normalization (ALP < 1 × ULN). Patients with similar disease stage at baseline were compared between mAChR3inh+ and mAChR3− based on ultrasound with absence of fibrosis classified as early stage and clear signs of fibroses classified as advanced stage. *p* = Analysis was done using Fisher’s exact test, n.s. = not significant.

PBC: In the PBC cohort, 38.5% (5/13) of mAChR3inh+ patients showed an inadequate response to UDCA therapy according to Paris I criterion, while only 16.2% (24/148) of mAChR3− patients showed inadequate treatment response rate. Among the mAChR3inh+ patients of whom follow up data on treatment response were available, 53.8% (7/13) of the patients were in advanced stage disease, whereas 46.2% (6/13) of the patients were in early stage based on ultrasound imaging (Table 1). Patients with mAChR3inh+ auto-ab tended to have a lower response rate to UDCA therapy than patients within the mAChR3 group: Only 50% (3/6) of mAChR3inh+ patients in early stage achieved an adequate response to therapy versus 87.5% (91/104) mAChR3− patients in early stage (*p* = 0.039) according to Paris I criterion. Within the mAChR3inh+ group in advanced stage an adequate treatment response was achieved in 71.4% (5/7) patients compared to 75.6% (31/41) patients in the mAChR3− group (Figure 4, Table S2). 

Further comparisons of response rates between mAChR3inh+ and mAChR3− patients with respect to disease stage according to Paris II, Rochester, Rotterdam and ALP normalization showed a tendency towards lower treatment response rates in mAChR3inh+ patients in early stage which did not achieve statistical significances (Figure 4, Table S2). However, this analysis needs to be regarded with caution given the low number of cases within each subgroup. 

PSC: In comparison, PSC patients with mAChR3inh+ab showed similar levels of bilirubin, ALP and GGT as compared to PSC patients without mAChR3inh+ auto-ab under the treatment with UDCA, when administered as off-label therapy (Table S1).

### 3.5. Relation between the Prevalence of Functional mAChR3inh+ Auto-ab and Long-Term Prognosis in PBC and PSC 

**Figure 5:**
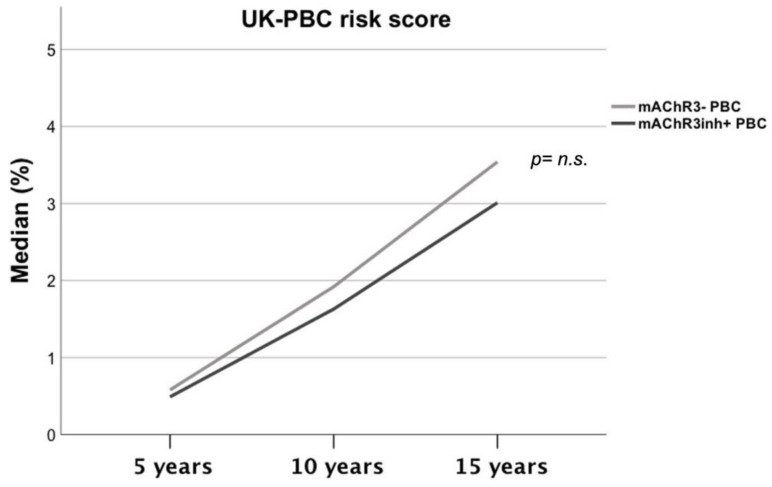
Comparison of UK-PBC risk score according to the functional mAChR3− status, *p* = Analysis was done using Mann–Whitney U Test.

PBC: According to the UK-PBC Score there were no differences for the risk of liver failure in patients with mAChR3inh+ compared to mAChR3− patients (Figure 5).

**Figure 6:**
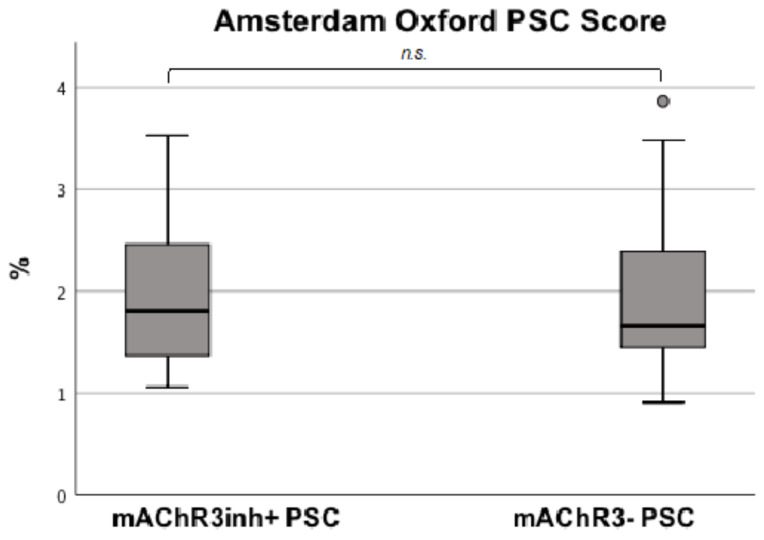
Comparison of Amsterdam-Oxford PSC score according to functional mAChR3− status. Analysis was done using Mann-Whitney U Test.

PSC: The Amsterdam–Oxford Score, which is used to estimate the probability of a transplant free survival, did not significantly differ between mAChR3inh+ and mAChR3− PSC patients (Figure 6).

### 3.6. Clinical Outcome in Correlation with Prevalence of Functional mAChR3 Auto-ab in PBC and PSC

PBC: Long-term follow-up evaluation showed significantly higher number of patients with liver cirrhosis in mAChR3inh+ PBC patients as compared to mAChR3− PBC patients (64%, 29/45 vs. 42%, 140/333, *p* = 0.019). In addition, significantly more patients developed ascites and varices within the mAChR3inh+ group than in the mAChR3− group (46.7%, 7/15 vs. 17.8%, 32/180, *p* = 0.014) (Table 3). Cholangiocellular carcinoma (CCC) and hepatocellular carcinoma (HCC) did not occur more frequently in patients with mAChR3inh+ as compared to mAChR3− patients (Table 3). With a 10-year transplant free survival of 95.1% in mAChR3− PBC patients versus 91% in mAChR3inh+ PBC patients, there was no significant difference in necessity for liver transplantation (data not shown).

PSC: In the PSC cohort cirrhosis as well as ascites were the most common long-term complications, which tended to occur more often in mAChR3inh+ patients, although this finding was not significant. The incidence of HCC and CCC was similar in mACHR3inh+ and mAChR3− patients (Table 3). In the PSC cohort, the median transplant-free survival time for the mAChR3inh+ PSC patients (*n* = 43) was 12 years and 5 months, and for the mAChR3− PSC patients (*n* = 67) it was 12 years and 3 months. 

In summary, the functional mAChR3 auto-ab status was not predictive for the long-term transplant free survival, neither in the PBC nor in the PSC cohort. 

## 4. Discussion

In this study the prevalence and potential relevance of inhibitory auto-ab against the mAChR3 was evaluated in large cohorts of German PBC patients. Furthermore, this is the first study to evaluate the presence of functional auto-ab against mAChR3 in a large cohort of PSC patients.

Our study provides several important findings: The prevalence of inhibitory auto-ab in PSC (33.6%) and in PBC (11.2%) was significantly higher than in healthy controls (2.5%). In PBC, but not PSC patients, the presence of mAChR3inh+ was associated with increased levels of biochemical cholestasis and hepatic inflammation. In PBC we observed a tendency towards decreased treatment response to standard UDCA therapy according to the well-established criteria in mAChR3inh+ patients in early stage. Moreover, mAChR3inh+ PBC patients experienced higher rate of liver cirrhosis at long-term follow-up. However, the presence of mAChR3inh+ auto-ab was not predictive for the long-term transplant free survival, neither in PBC nor in PSC. Notably, since only a few patients had mAChR3stim+ auto-ab, we focused our further analyses on the clinical relevance of mAChR3inh+ auto-ab, which were found in a larger proportion of patients.

Auto-ab against mAChR3 in PBC was first described by Berg et al. in 50 patients, testing patients’ sera against an immunodominant peptide of the mAChR3 by ELISA [24]. Tsuboi et al. examined PBC patients for the presence of mAChR3 auto-ab. They found auto-ab in up to 90% of the PBC cohort [23]. This study also included 10 patients with PSC of whom 60% (6/10) were positive for auto-ab against mAChR3. However, in this study no functional data of the auto-ab were investigated. Compared to previous studies [23,24] the number of patients with mAChR3 auto-ab is rather small, which is most likely due to our measurement method as we examined the presence of functionally active auto-ab and not the overall level of mAChR3 auto-ab.

While laboratory findings in our study did not differ between mAChR3inh+ PSC and mAChR3− PSC, there were significant differences in baseline parameters according to the individual auto-ab status within the PBC cohort. MAChR3inh+ PBC patients showed higher baseline levels of transaminases, GGT-, ALP- and bilirubin levels. Moreover, mAChR3inh+ patients tended to be in a more advanced disease stage at first presentation compared to mAChR3− patients and showed more frequently an inadequate treatment response after one year of UDCA with reaching statistically significance according to Paris I criterion. Importantly, patients with mAChR3inh+ showed a higher rate in experiencing liver cirrhosis at follow-up. Compared to our recently published work by Mayer et al. [27] we were able to show in the present study a correlation between the presence of mAChR3inh+ auto-ab and clinical presentation. This could be due to the fact that the patients in this study were recruited from two large tertiary care centers and probably already showed a more advanced disease stage at first presentation. Therefore, a selection bias cannot be ruled out. 

Few limitations of the present study need to be acknowledged. A systematic evaluation of all biochemical parameters in every study participant was not available due to the retrospective character of this study. Especially the number of PBC patients with follow up data at 12 months after treatment initiation was small.

Similar to PBC, also in the PSC cohort cirrhosis as well as ascites tended to occur more often in mAChR3inh+ patients, although this finding was not significant.

However, it remains unclear whether the relation between mAChR3inh+ auto-ab and the disease severity at baseline in PBC is associative or whether a causal mechanistic link exists between the presence of mAChR3inh+ auto-ab and the more severe disease state at first diagnosis and follow-up biochemical parameters.

Previous studies showed a lower biochemical treatment response in patients with higher baseline bilirubin, ALP or AST levels [29]. Thus, the association between mAChR3inh+ auto-ab and rather lower treatment response may result from more severe disease state at first presentation in mAChR3inh+ PBC patients.

Moreover, elevated ALP is predictive for liver transplantation in PBC [34]. In the present study, 13.3% of the mAChR3inh+ PBC patients underwent liver transplantation compared to 5.9% of the mAChR3− patients. However, this trend did not reach statistical significance. Thus, the prognostic value of mAChR3inh+ auto-ab with regard to the necessity of liver transplantation as well as long-term survival still remains to be established in further independent cohorts.

Since the pathogenesis of PBC and PSC is still unclear and an autoimmune component is increasingly proposed, many studies have been performed to identify a possible immunological cause [35,36,37]. 

PBC is characterized by disease-specific anti-mitochondrial autoantibodies (AMAs) and autoreactive T cells due to loss of immunotolerance to the pyruvate dehydrogenase complex. AMAs can be detected in up to 95% of PBC patients [28,35]. However, the pathogenetic relevance of these auto-ab still remains unclear. So far, no causal link between the presence of the auto-ab and the development of PBC has been demonstrated. Notably, inducing aberrant PDC-E2 expression in a transgenic mouse did not cause the disease in animal models [38]. Moreover, reduction in the auto-ab titer of AMA did not improve the disease manifestation [18,38,39]. Thus, there is an ongoing debate whether AMAs are pathogenic to the disease or rather the consequence of autoimmune reactions [10].

One explanation is the ubiquitous expression of the antigen of AMA, PDC-E2, in the human organism, and thus, the presence of AMAs does not explain why the PBC mainly manifests itself in the biliary tract [38]. Unlike AMAs, the mAChR3 is expressed in BECs but not in hepatocytes and, therefore, mAChR3− associated pathogenesis could explain organ specificity of PBC.

In patients suffering from PSC, a high frequency of biliary epithelial cell antibodies (BEC-ab) is present, which promote the recruitment of inflammatory cells via production of cytokines and chemokines [40]. However, with regard to patients with PSC, our data showed no correlation between mAChR3 and severity of the disease. This suggests that dysfunctional signaling pathways of mAChR3 are mainly relevant in PBC, but not in PSC, as PSC may have other underlying pathomechanisms.

One possible reason could be the heterogeneity of BECs with regard to morphology, biochemical and functional characteristics [41,42]. In particular, BECs show increasingly higher degree of differentiation with increasing size of ducts branching [4,41]. Han et al. also showed that there is a difference in the function of small and large BECs. While the functional signaling in large bile ducts is mainly based on cAMP-dependent pathways, the small bile ducts act mainly via IP3/calcium-dependent signaling [41]. There are only few data on the expression of mAChR3 in the development of bile ducts from small to large bile ducts. It is well known that in patients with PBC the small bile ducts and in patients with PSC mainly the larger bile ducts are affected. Notably, acetylcholine increases the intracellular IP3/calcium concentration via binding to the mAChR3. According to Han et al., in small bile ducts cAMP-dependent signaling is of rather minor relevance as compared to large bile ducts [41]. This could explain the greater influence of mACh3Rinh+ auto-ab on bicarbonate secretion and therefore for disease progression in patients with PBC but not with PSC.

MAChR3 dependent signaling pathways have been suggested to be involved in the development of cholangiocarcinoma (CCC) [43,44] and colorectal adenocarcinoma (CRC) [45]. We found no evidence that mAChR3inh+ auto-ab patients may be at lower risk of developing CCC or CRC, although in vitro data from BEC cultures suggest that blocking mAChR reduces tumor growth [45].

Taken together, our findings contribute to our understanding of the potential role of mAChR3inh+ auto-ab in chronic inflammatory diseases. The association of mAChR3inh+ auto-ab and laboratory characteristics and treatment response to standard UDCA therapy severity in PBC support the hypothesis that dysfunctional mAChR3−mediated signaling may be involved in the pathogenesis of PBC, but less likely in PSC. However, additional mechanistic studies are required to dissect the causal role of mAChR3inh+ auto-ab in the development of chronic biliary inflammation. Further studies should also explore the prognostic value of mAChR3inh+ auto-ab in PBC patients to identify patients at high-risk of UDCA non-response and disease progression.

## Figures and Tables

**Table 1:** Baseline characteristics according to functional mAChR3 auto-ab status of the PBC cohort.

Parameter	mAChR3inh+ PBC	*n*	mAChR3− PBC	*n*	*p*
mAChR3inh+/− status in patients (%)	11.8	46/389	88.2	343/389	-
Sex: female (%)	91.3	42/46	92.1	316/343	ns
Age at time of diagnosis (years)	58(18)	31/ 46	55 (15)	243/341	ns
Age > 40 (%)	87.0	27/31	92.5	225/243	ns
Age < 40 (%)	13.0	4/31	7.2	18/243	ns
AMA	75.0	24/32	75.4	193/256	ns
Elastography (kPa) *	8.5 (2.8)	11/46	8.0 (4.7)	157/343	ns
Clear signs of fibrosis based on ultrasound (%) **	65.0	13/20	49.6	120/242	ns
Advanced histological stage (%) ***	63.6	7/11	38.7	41/106	ns
Sicca syndrome (%)	17.9	5/28	15	39/260	ns
Autoimmune thyreoiditis (%)	14.8	4/27	9.6	25/260	ns
Autoimmune hepatitis (%)	14.3	4/28	17.3	45/260	ns
Rheumatic disorders (%)	3.7	1/27	17.3	45/260	ns
Alanine aminotransferase (ULN)	1.7 (1.9)	45	1.3 (1.2)	329	0.01
Aspartate aminotransferase (ULN)	1.3 (1.5)	46	0.9 (0.7)	327	0.002
Alkaline phosphatase (ULN)	4.0 (2.9)	46	1.8 (1.9)	332	<0.001
Gamma-glutamyltransferase (ULN)	5.4 (9.8)	45	3.4 (5.2)	332	<0.001
Bilirubin (ULN)	0.7 (0.8)	46	0.4 (0.4)	311	<0.001
Albumin (g/dl)	4.3 (0.5)	23	4.4 (0.5)	188	ns
INR	1.1 (0.2)	6	1.0 (0.1)	67	ns
Platelet count (/nl)	235 (97)	37	260 (79)	283	ns

Median (IQR), *p* = Analysis was done using Mann–Whitney U Test and Fisher´s exact test. * liver stiffness in kPa at first elastography screening, ** diagnosis was made according to findings in first ultrasound screening, *** including histological stage 3 and 4 according to Ludwig et al. [32], ns = not significant.

**Table 2:** Baseline characteristics according to functional mAChR3 auto-ab status of the PSC cohort.

Parameter	mAChR3inh+ PSC	*n*	mAChR3− PSC	*n*	*p*
mAChR3inh+/− status in patients (%)	38.1	45/118	61.9	73/118	*-*
Sex: male (%)	73.3	33/45	57.5	42/73	ns
Age at time of diagnosis (years)	37 (15)	42/45	33 (19)	65/73	ns
Elastography (kPa) *	7.9 (8.7)	7/45	8.75 (6.25)	8/73	ns
Clear signs of fibrosis based on ultrasound (%) **	50.0	19/38	50.9	28/55	ns
Advanced histological stage (%) ***	20.6	7/34	28.4	19/67	ns
Ulcerative Colitis (%)	51.1	23/45	57.5	42/73	ns
Crohn’s disease (%)	15.6	7/45	9.6	7/73	ns
Autoimmune hepatitis (%)	22.2	10/45	2.7	2/73	0.001
Alanine aminotransferase (ULN)	1.93 (1.89)	27	2.15 (2.53)	46	ns
Aspartate aminotransferase (ULN)	1.06 (0.75)	28	1.06 (1.31)	45	ns
Alkaline phosphatase (ULN)	3.06 (4.36)	26	2.78 (3.08)	46	ns
Gamma-glutamyltransferase (ULN)	4.93 (5.26)	26	3.77 (6.24)	46	ns
Bilirubin (ULN)	0.7 (0.57)	13	0.63 (0.51)	26	ns
Albumin (g/dl)	4.3 (1)	15	4.4 (0.6)	37	ns
Platelet count (/nl)	278 (88.8)	27	241 (114.9)	45	ns

Median (IQR), * liver stiffness in kPa at first elastography screening, ** diagnosis was made according to findings in first ultrasound screening, *** including histological stage 3 and 4 according to Ludwig et al. [32], *p* = Analysis was done using Mann–Whitney U Test and Fisher’s exact test.

**Table 3:** Long-term follow-up data according to functional mAChR3 auto-ab status of the PBC and PSC cohort.

**Parameter**	**mAChR3inh+ PBC**	**n**	**mAChR3− PBC**	**n**	***p***
Cirrhosis (%)	64.4	29/45	42	140/333	0.019
Ascites, Varices (%)	46.7	7/15	17.8	32/180	0.014
Carcinoma (HCC/CCC) (%)	5.0	1/20	0.9	2/225	ns
Liver transplant (%)	13.3	6/45	5.9	20/339	ns
**Parameter**	**mAChR3inh+ PSC**	**n**	**mAChR3− PSC**	**n**	***p***
Cirrhosis (%)	36.8	14/38	31.6	18/57	ns
Ascites (%)	15.8	6/38	7.0	4/57	ns
Cholangiocarcinoma (HCC/CCC) (%)	4.4	2/45	6.8	9/73	ns
Liver transplant (%)	46.0	29/63	50.8	63/124	ns

*p* = Analysis was done using Fisher’s exact test. HCC (hepatocellular carcinoma), CCC (Cholangiocellular carcinoma), ns = not significant.

## Supplementary Materials

The following supporting information can be downloaded at: https://www.mdpi.com/article/10.3390/jcm11030681/s1, Table S1: Laboratory of PSC patients 12 months after treatment initiation, Table S2: Treatment response after 12 months of UDCA therapy in mAChRinh+ and mAChR− PBC patients according to disease stage at baseline.
