# Supplementary material for: Evaluation of Inhibitory Antibodies against the Muscarinic Acetylcholine Receptor Type 3 in Patients with Primary Biliary Cholangitis and Primary Sclerosing Cholangitis
Source: J Clin Med. 2022 Jan 28;11(3):681. doi: 10.3390/jcm11030681 (PMC8836427; doi:10.3390/jcm11030681)
Supplement: Supplementary file 1 [file jcm-11-00681-s001.zip › jcm-1518789-supplementary.pdf]

Journal of
Clinical Medicine

Supplementary Materials

Table S1. Laboratory parameters of PSC patients 12 months after treatment initiation.

Parameter	mAChR3+ PSC	*n*	mAChR3− PSC	*n*	*p*
ALT (ULN)	0.73 (1.46)	16	1.10 (2.57)	29	ns
AST (ULN)	0.62 (1.00)	15	0.94 (0.90)	29	ns
ALP (ULN)	1.42 (2.03)	16	1.52 (2.46)	29	ns
GGT (ULN)	1.23 (1.83)	15	1.73 (3.01)	28	ns
Bilirubin (ULN)	0.58 (0.67)	15	0.83 (0.67)	25	ns

Median (IQR).

Table S2. Treatment response after 12 months of UDCA therapy in mAChRinh+ and mAChR- PBC patients according to disease stage at baseline.

			mAChR3inh+	*n*	mAChR3−	*n*	*p*
Paris I							
	early	adequate (%)	50	3/6	87.5	91/104	0.039
		inadequate (%)	50	3/6	12.5	13/104	
	advanced	adequate (%)	71.4	5/7	75.6	31/41	ns
		inadequate (%)	28.6	2/7	24.4	10/41	
Paris II							
	early	adequate (%)	50	3/6	72	77/107	ns
		inadequate (%)	50	3/6	28	30/107	
	advanced	adequate (%)	42.9	3/7	38.1	16/42	ns
		inadequate (%)	57.1	4/7	61.9	26/42	
Rochester							
	early	adequate (%)	66.6	4/6	86.5	90/104	ns
		inadequate (%)	33.3	2/6	13.5	14/104	
	advanced	adequate (%)	71.4	5/7	70.1	29/41	ns
		inadequate (%)	28.6	2/7	29.3	12/41	
Rotterdam							
	early	adequate (%)	75	3/4	98.9	90/91	ns
		inadequate (%)	25	1/4	1.1	1/91	
	advanced	adequate (%)	85.7	6/7	91.9	34/37	ns
		inadequate (%)	14.3	1/7	8.1	3/37	
ALP Normalization							
	early	adequate (%)	33.3	2/6	45.7	48/105	ns
		inadequate (%)	66.6	4/6	54.3	57/105	
	advanced	adequate (%)	14.3	1/7	28.6	12/42	ns
		inadequate (%)	86.7	6/7	71.4	30/42	

p = Analysis was done using Fisher's exact test.
